# Inducing Ferroptosis to Enhance Radiotherapy in Head and Neck Cancer: Mechanisms, Radiosensitization Strategies, and Normal Tissue Considerations

**DOI:** 10.3390/cells15090812

**Published:** 2026-04-29

**Authors:** Jaewang Lee, Jong-Lyel Roh

**Affiliations:** 1 Department of Otorhinolaryngology-Head and Neck Surgery, CHA Bundang Medical Center, CHA University, Seongnam 13496, Republic of Korea; 2 Logsynk, Seoul 06153, Republic of Korea; 3 Department of Biomedical Science, General Graduate School, CHA University, Pocheon 11160, Republic of Korea

**Keywords:** ferroptosis, radiotherapy, head and neck cancer, radiosensitization, lipid peroxidation, normal tissue protection

## Abstract

**Highlights:**

**What are the main findings?**

- Radiotherapy can induce ferroptosis through ROS-driven lipid peroxidation and antioxidant disruption.
- Ferroptosis susceptibility strongly influences radiosensitivity and radioresistance in head and neck cancers.
- Targeting lipid metabolism, iron homeostasis, and the SLC7A11–GPX4 axis enhances radiation-induced ferroptosis.

**What are the implications of the main findings?**

- Ferroptosis induction represents a promising strategy to radiosensitize resistant head and neck tumors.
- Tumor-selective ferroptosis modulation may widen the therapeutic ratio of radio-therapy.
- Integrating ferroptosis biology with radiotherapy and immunotherapy could improve treatment outcomes.

**Abstract:**

Ferroptosis is an iron-dependent form of regulated cell death characterized by lipid peroxidation and failure of cellular antioxidant defenses. Increasing evidence indicates that ferroptosis contributes to the biological effects of radiotherapy and influences both tumor radiosensitivity and normal tissue injury. Because radiotherapy is a central treatment modality for many head and neck cancers, understanding how ferroptosis interacts with radiation responses has important translational implications. Ionizing radiation can induce ferroptosis through reactive oxygen species generation, disruption of glutathione metabolism, suppression of the SLC7A11–GSH–GPX4 antioxidant axis, and remodeling of membrane lipid composition. Conversely, tumor cells frequently develop radioresistance by reinforcing ferroptosis-suppressive pathways, including enhanced cystine transport, lipid desaturation, and metabolic adaptation. In head and neck cancers such as head and neck squamous cell carcinoma, nasopharyngeal carcinoma, oral squamous cell carcinoma, and thyroid malignancies, experimental studies show that modulation of ferroptosis significantly alters radiation response. Strategies that promote ferroptosis—including inhibition of antioxidant defenses, targeting of lipid metabolism, and modulation of iron homeostasis—have demonstrated radiosensitizing effects in preclinical models. However, ferroptosis may also contribute to radiation-induced normal tissue injury, particularly in oxidative stress-sensitive organs such as the salivary glands. This review summarizes the molecular basis of ferroptosis in radiotherapy, examines its role in radiosensitivity and radioresistance in head and neck cancers, and discusses therapeutic strategies to exploit ferroptosis while minimizing normal tissue toxicity.

## 1. Introduction

Ferroptosis has emerged as a biologically distinct form of regulated cell death driven by iron-dependent lipid peroxidation and catastrophic loss of membrane redox homeostasis [1,2]. Unlike apoptosis, ferroptosis is primarily defined by the oxidative destruction of polyunsaturated phospholipids, with characteristic involvement of labile iron, reactive oxygen species (ROS), and failure of antioxidant defense systems centered on SLC7A11, glutathione (GSH), and glutathione peroxidase 4 (GPX4) [3,4]. Because ionizing radiation (IR) directly generates ROS and perturbs redox balance, the interface between radiotherapy (RT) and ferroptosis has drawn increasing attention as a mechanistic explanation for part of RT-induced tumor cell killing and as a potential therapeutic opportunity for radiosensitization [5,6,7,8,9,10].

RT remains a cornerstone of modern cancer treatment and is used in the management of a large proportion of patients with malignant disease [11]. Historically, the anti-tumor effects of RT have been interpreted mainly through DNA damage, especially double-strand breaks, mitotic catastrophe, and apoptosis [12]. However, recent work has broadened this paradigm by showing that RT also remodels lipid metabolism, promotes lipid peroxidation, and induces non-apoptotic cell death programs, including ferroptosis [6,9,10]. These observations support the view that ferroptosis is not merely an epiphenomenon of oxidative stress, but one of the regulated death pathways that can shape RT efficacy and resistance.

This concept is particularly relevant to head and neck cancer (HNC), in which RT plays a central therapeutic role across definitive, adjuvant, organ-preserving, and recurrent disease settings [13,14]. Yet local failure, intrinsic or acquired radioresistance, and treatment-limiting toxicity remain major clinical obstacles, especially in HNC [15]. The uploaded HNC-focused literature indicates that ferroptosis is increasingly implicated in these problems at multiple levels, including tumor metabolism, antioxidant adaptation, iron handling, lipid remodeling, and immune interaction [16,17,18]. In head and neck squamous cell carcinoma (HNSCC), recent studies have shown that RT-induced ferroptosis can be enhanced by glutamine blockade combined with CD47 inhibition, by statins, by MitoTam, and by targeting DDR1 signaling, whereas prognostic and diagnostic models have also linked ferroptosis- and RT-related gene signatures to clinical heterogeneity [19,20,21,22,23,24]. Nasopharyngeal carcinoma (NPC) provides especially strong support for a ferroptosis-based radiosensitization framework. The studies compiled in the search file show that IR itself can induce ferroptotic features in NPC cells, whereas radioresistance can be driven by several ferroptosis-suppressive programs, including SLC7A11 upregulation, METTL3-mediated stabilization of SLC7A11 transcripts, CD38-mediated protection of SLC7A11 protein through TRIM21 competition, AGT/HIF-1α/HILPDA-driven lipid droplet accumulation, HOTAIRM1/FTO/YTHDC1/CD44 signaling, and loss of TXNIP or PCK2 [25,26,27,28,29,30,31]. Conversely, radiosensitization has been achieved experimentally through GSTM3, ACSL4 acetylation, circADARB1-targeting nanocarriers, SOD2 depletion, and other ferroptosis-promoting interventions, further underscoring the mechanistic importance of ferroptosis in NPC RT response [32,33,34,35,36]. Together, these data suggest that ferroptosis is not peripheral to NPC radiobiology but rather increasingly appears to be one of its central actionable vulnerabilities.

The potential relevance of ferroptosis extends beyond conventional HNSCC and NPC. In oral squamous cell carcinoma (OSCC), hyperbaric oxygen and astaxanthin have both been reported to enhance RT-associated ferroptotic responses, while cold atmospheric plasma irradiation has been linked to mixed death signaling that includes ferroptosis-related mechanisms [37,38,39]. In thyroid malignancies represented within the uploaded dataset, CHAC1 promoted ferroptosis and enhanced radiosensitivity in thyroid carcinoma, whereas YTHDF2-mediated stabilization of SREBF1 drove lipid metabolic remodeling and ferroptosis-associated radioresistance in anaplastic thyroid carcinoma [40,41]. Although these disease entities differ biologically and clinically, the accumulating evidence points to a recurring theme: RT response in head and neck-region malignancies is strongly influenced by the balance between lipid peroxidation pressure and ferroptosis defense systems.

At the same time, ferroptosis in the context of RT must be viewed as a double-edged sword. The same oxidative and lipid-peroxidative processes that may improve tumor control can also contribute to radiation injury in adjacent normal tissues [6]. This issue is particularly important in HNC, where RT commonly affects salivary glands, oral mucosa, swallowing structures, skin, and other functionally critical tissues [42]. The uploaded reviews on RT and ferroptosis emphasize that ferroptosis can participate not only in tumor suppression but also in radiation injury and late normal-tissue toxicity [6,7,8]. Consistent with this concern, the search file includes a recent review on radiation-induced salivary gland dysfunction highlighting ferroptosis as an emerging mechanism relevant to xerostomia and unmet supportive care needs in patients with HNC [43]. Therefore, any strategy designed to induce ferroptosis for radiosensitization in HNC must also address tumor selectivity and normal tissue protection.

Although many of the molecular mechanisms linking radiotherapy and ferroptosis have been described across diverse cancer types, focusing specifically on head and neck cancer is clinically and biologically justified. RT is a cornerstone of HNC treatment across multiple disease settings, and outcomes are strongly influenced by radioresistance and treatment-related toxicity in anatomically and functionally critical regions. In addition, emerging HNC-specific studies have identified distinct ferroptosis-regulatory pathways, metabolic adaptations, and therapeutic vulnerabilities that are not fully captured in pan-cancer analyses. Therefore, a disease-focused synthesis is necessary to translate general ferroptosis biology into clinically relevant strategies for head and neck oncology. While previous landmark reviews have established ferroptosis as a therapeutic vulnerability in cancer, particularly in overcoming resistance to conventional therapies [44], their focus has largely remained on general oncologic contexts. In contrast, this review specifically integrates ferroptosis with RT biology in HNC, highlighting its dual role in tumor radiosensitization and normal tissue response.

We first summarize the core molecular basis of ferroptosis most relevant to RT response. We then examine how RT induces ferroptosis across cancers and how ferroptosis contributes to radiosensitivity or radioresistance. Building on that framework, we focus on the available HNC-specific evidence, spanning HNSCC, NPC, OSCC, and thyroid malignancies, to evaluate how ferroptosis induction could be exploited to enhance RT efficacy. Finally, we address the opposite but equally important question of whether understanding ferroptosis may also help reduce RT-induced tissue and organ injury in the head and neck field. Collectively, the current literature supports ferroptosis not only as a biologically meaningful consequence of irradiation, but also as a promising therapeutic axis for improving the therapeutic ratio of RT in HNC.

## 2. Core Molecular Basis of Ferroptosis Relevant to Radiotherapy

Ferroptosis is a form of regulated cell death characterized by iron-dependent lipid peroxidation and the collapse of cellular antioxidant defenses that normally prevent oxidative damage to membrane phospholipids [1,45]. Unlike apoptosis or necroptosis, ferroptosis is driven primarily by the accumulation of lipid hydroperoxides in polyunsaturated fatty acid (PUFA)-containing phospholipids within cellular membranes [18,46]. When these oxidized lipids exceed the detoxifying capacity of antioxidant systems, membrane integrity is lost, resulting in catastrophic oxidative cell death [6,8,10]. Because IR produces abundant ROS and perturbs cellular redox balance, several molecular pathways that govern ferroptosis are directly relevant to the biological response to RT.

### 2.1. Iron Metabolism and the Generation of Lipid Peroxidation

Iron availability is a central determinant of ferroptosis. Labile ferrous iron (Fe^2+^) participates in Fenton chemistry, generating highly reactive hydroxyl radicals that initiate lipid peroxidation reactions within membrane phospholipids [18,47]. Cellular iron homeostasis is therefore tightly regulated through coordinated control of iron uptake, storage, and export. Transferrin receptor-mediated iron import, ferritin-mediated storage, and ferroportin-mediated export collectively determine the intracellular iron pool available for redox reactions [48].

Arachidonic acid (AA), a major PUFA in membrane phospholipids, plays a central role in ferroptosis due to its high susceptibility to peroxidation [49]. The presence of bis-allylic hydrogen atoms in AA facilitates hydrogen abstraction by ROS, initiating lipid radical formation and propagation of lipid peroxidation chains. Enzymatic incorporation of AA into phospholipids by Acyl-CoA synthetase long-chain family member 4 (ACSL4) and lysophosphatidylcholine acyltransferase 3 (LPCAT3) further enhances ferroptosis sensitivity by increasing the availability of peroxidation-prone substrates [50,51]. In addition, ascorbic acid can influence iron-dependent lipid peroxidation through its redox activity. Under certain conditions, ascorbate reduces ferric iron (Fe^3+^) to ferrous iron (Fe^2+^), thereby promoting Fenton reactions and enhancing the generation of hydroxyl radicals. This pro-oxidant activity can amplify lipid peroxidation when antioxidant defenses are overwhelmed, contributing to ferroptotic cell death.

Disruption of these regulatory mechanisms can increase susceptibility to ferroptosis. Ferritin degradation through ferritinophagy releases stored iron into the cytosolic labile iron pool, thereby amplifying ROS-driven lipid peroxidation [52,53,54]. Conversely, enhanced iron sequestration can suppress ferroptotic death by limiting the catalytic iron necessary for lipid oxidation. Importantly, IR has been reported to alter iron metabolism through multiple mechanisms, including modulation of ferritin expression, increased ROS-mediated ferritin degradation, and altered mitochondrial iron handling [55]. These processes may increase the availability of catalytic iron and thereby promote ferroptosis following irradiation [6,7,9].

At the biochemical level, lipid peroxidation proceeds through a radical chain reaction initiated by Fe^2+^-dependent Fenton chemistry. Ferrous iron reacts with hydrogen peroxide to generate highly reactive hydroxyl radicals (•OH), which abstract hydrogen atoms from bis-allylic positions of PUFAs within membrane phospholipids [18]. This process generates lipid radicals (L•), which rapidly react with molecular oxygen to form lipid peroxyl radicals (LOO•). These radicals further propagate the reaction by abstracting hydrogen from adjacent lipids, leading to the accumulation of lipid hydroperoxides (LOOH) and cyclic endoperoxide intermediates.

These oxidized lipid species are highly cytotoxic because they disrupt membrane architecture, increase membrane permeability, and impair the function of membrane-associated proteins [18]. In particular, lipid hydroperoxides and endoperoxides can decompose into reactive aldehydes and electrophilic fragments, which further damage proteins and nucleic acids [45]. When the detoxification capacity of GPX4 and related antioxidant systems is exceeded, uncontrolled accumulation of these lipid peroxidation products results in irreversible membrane damage and ferroptotic cell death.

### 2.2. Lipid Metabolism and the Formation of Peroxidation-Prone Phospholipids

Ferroptosis critically depends on the composition of cellular membrane lipids, particularly the abundance of PUFA-containing phospholipids that serve as substrates for lipid peroxidation [56]. Two enzymes play central roles in generating these susceptible lipid species. ACSL4 converts long-chain PUFAs into acyl-CoA derivatives, while LPCAT3 incorporates these PUFA moieties into membrane phospholipids [50]. This enzymatic pathway enriches cellular membranes with oxidizable phospholipids, thereby increasing ferroptosis sensitivity [6,8].

In contrast, monounsaturated fatty acids (MUFAs) can antagonize ferroptosis by replacing PUFAs in membrane phospholipids and reducing susceptibility to peroxidation [45,49]. This protective mechanism involves enzymes such as ACSL3 and stearoyl-CoA desaturase 1 (SCD1), which promote MUFA synthesis and incorporation into phospholipids. Tumor cells frequently exploit this lipid remodeling strategy to resist oxidative damage and ferroptosis. Notably, RT can influence lipid metabolism by increasing ROS-dependent lipid oxidation and altering the activity of lipid metabolic enzymes [57]. Consequently, the lipid composition of tumor cell membranes represents an important determinant of whether irradiation ultimately leads to ferroptotic cell death or adaptive survival [6,10].

### 2.3. The SLC7A11–GSH–GPX4 Antioxidant Defense Axis

The most well-characterized protective mechanism against ferroptosis is the GSH-dependent antioxidant system centered on the cystine/glutamate antiporter system Xc^−^, GSH, and GPX4 [58,59]. System Xc^−^, composed of the transporter subunit SLC7A11 and the regulatory component SLC3A2, imports extracellular cystine in exchange for intracellular glutamate [60]. Once inside the cell, cystine is reduced to cysteine and used for GSH synthesis [61]. GPX4 then uses GSH as a cofactor to detoxify phospholipid hydroperoxides, converting them into non-toxic lipid alcohols and thereby preventing ferroptotic membrane damage [59,62]. Inhibition of any component of this pathway—SLC7A11 activity, GSH synthesis, or GPX4 enzymatic function—can trigger ferroptosis by allowing lipid peroxides to accumulate. Several studies have shown that RT can influence this axis through ROS-mediated GSH depletion and transcriptional repression of SLC7A11 [63]. Activation of the ATM kinase following DNA damage has been reported to suppress SLC7A11 expression, thereby lowering intracellular cystine uptake and sensitizing cells to ferroptosis [25,26]. Tumor cells frequently upregulate SLC7A11 or GPX4 as adaptive responses to oxidative stress, which can contribute to radioresistance. Accordingly, pharmacologic inhibition of system Xc^−^ or GPX4 has emerged as a promising strategy to enhance RT-induced ferroptosis and improve tumor control.

### 2.4. GPX4-Independent Ferroptosis Defense Systems

Although the SLC7A11–GSH–GPX4 axis is central to ferroptosis regulation, additional antioxidant pathways can suppress lipid peroxidation independently of GPX4. One such pathway involves ferroptosis suppressor protein 1 (FSP1), which reduces coenzyme Q_10_ (CoQ_10_) to its antioxidant form, ubiquinol, thereby preventing lipid radical propagation within cellular membranes [64,65,66]. This FSP1–CoQ_10_ system functions parallel to GPX4 and can compensate for GPX4 inhibition in certain cellular contexts. Other GPX4-independent ferroptosis defense mechanisms include the GTP cyclohydrolase 1 (GCH1)–tetrahydrobiopterin (BH_4_) pathway and mitochondrial dihydroorotate dehydrogenase (DHODH) [67,68]. The BH_4_ system acts as a potent lipid antioxidant and can directly inhibit lipid peroxidation, whereas mitochondrial DHODH contributes to ferroptosis suppression within mitochondrial membranes. These pathways highlight the complexity of ferroptosis regulation and suggest that tumor cells may rely on multiple redundant antioxidant systems to resist oxidative death. Importantly, some of these pathways have been implicated in RT resistance, suggesting that targeting alternative ferroptosis defense systems may broaden radiosensitization strategies.

### 2.5. Crosstalk Between Radiation-Induced Oxidative Stress and Ferroptosis Signaling

RT generates ROS through water radiolysis and secondary redox reactions, producing superoxide anions, hydrogen peroxide, and hydroxyl radicals [69]. These reactive species not only damage DNA but also oxidize membrane lipids and disturb cellular redox homeostasis. As a result, several key biochemical conditions necessary for ferroptosis—including ROS accumulation, lipid peroxidation, and depletion of antioxidant capacity—can arise following irradiation [6,10]. However, the induction of ferroptosis by RT is not inevitable. Tumor cells often activate adaptive antioxidant responses, including NRF2-mediated transcriptional programs, increased SLC7A11 expression, and metabolic rewiring of lipid synthesis pathways [70,71,72]. These compensatory mechanisms may suppress ferroptosis and contribute to radioresistance. Conversely, when oxidative pressure overwhelms these protective systems, lipid peroxidation proceeds unchecked, leading to ferroptotic death. Thus, the balance between oxidative stress and ferroptosis defense pathways determines whether irradiation ultimately results in tumor cell survival or ferroptosis-mediated elimination.

Taken together, ferroptosis is regulated by an integrated network of iron metabolism, lipid remodeling, and antioxidant defense systems. Many of these molecular pathways intersect directly with the biological effects of IR, providing a mechanistic basis for the growing recognition that ferroptosis contributes to RT-induced tumor cell death. Understanding these core mechanisms is therefore essential for developing therapeutic strategies that deliberately induce ferroptosis to enhance RT efficacy while minimizing normal tissue toxicity. Similar integrative frameworks have been proposed in OSCC, where ferroptosis regulation is described as the interplay of antioxidant defense systems, lipid metabolism, iron homeostasis, and auxiliary suppressive pathways, reinforcing the conserved architecture of ferroptosis regulation across head and neck malignancies [73].

Mitochondria also undergo characteristic structural and functional alterations during ferroptosis. Electron microscopy studies have shown that ferroptotic cells exhibit condensed mitochondria with reduced volume, increased membrane density, and loss of cristae. Functionally, mitochondrial oxidative phosphorylation becomes impaired, accompanied by disruption of electron transport chain activity and increased production of mitochondrial ROS. This metabolic dysfunction further amplifies intracellular oxidative stress and promotes lipid peroxidation. In addition, lipid peroxidation of mitochondrial membranes compromises membrane integrity, leading to increased membrane permeability and bioenergetic failure. Unlike apoptosis, ferroptosis is not driven by classical mitochondrial outer membrane permeabilization or cytochrome c release. However, mitochondrial damage contributes to ferroptotic progression by facilitating the accumulation of reactive lipid species and redox-active metabolites, which further propagate oxidative injury within the cell.

## 3. How Radiotherapy Induces Ferroptosis Across Cancers

IR induces a broad spectrum of cellular stress responses, including DNA damage, metabolic perturbation, membrane oxidation, and inflammatory signaling. Although apoptosis and mitotic catastrophe have long been regarded as principal mediators of RT-induced tumor cell death, accumulating evidence indicates that ferroptosis is also an important component of the radiation response in multiple tumor types, particularly when oxidative stress and lipid peroxidation exceed the capacity of cellular antioxidant defenses [5,6,7,8,9,10].

**Figure 1:**
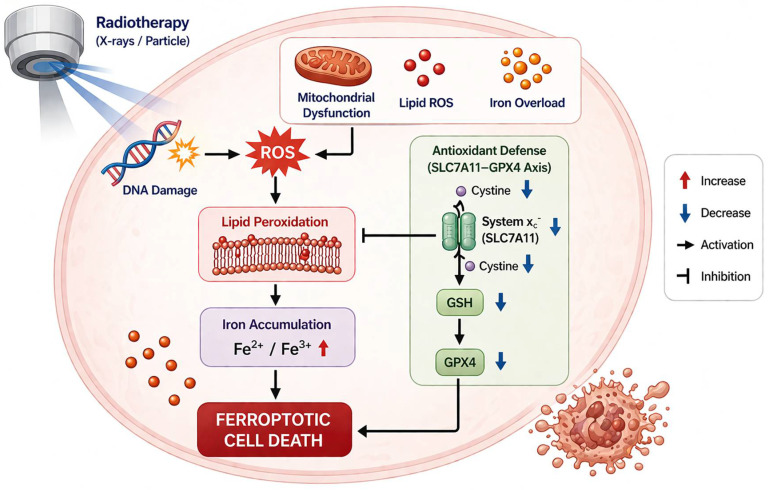
Molecular framework linking radiotherapy to ferroptosis. Radiotherapy induces ROS through DNA damage and mitochondrial dysfunction, promoting lipid peroxidation and intracellular iron accumulation. Increased labile iron further amplifies oxidative damage via iron-dependent reactions. At the same time, disruption of the SLC7A11–GSH–GPX4 antioxidant defense system impairs detoxification of lipid hydroperoxides. These combined processes lead to uncontrolled lipid peroxidation and ultimately ferroptotic cell death. Abbreviations: Fe^2+^, ferrous iron; Fe^3+^, ferric iron; GPX4, glutathione peroxidase 4; GSH, glutathione; ROS, reactive oxygen species; SLC7A11 (xCT), cystine/glutamate antiporter system component.

Rather than representing a uniform or inevitable consequence of irradiation, ferroptosis appears to arise when RT creates a permissive biochemical environment characterized by excessive ROS, depletion of GSH, impaired GPX4 activity, enrichment of oxidizable polyunsaturated phospholipids, and sufficient catalytic iron to drive lipid radical propagation (Figure 1). These events can occur in parallel and reinforce one another, thereby shifting irradiated tumor cells toward ferroptotic death rather than adaptive survival [5,6,9,10].

### 3.1. RT-Induced ROS Generation and Lipid Peroxidation as the Initiating Event

The most immediate connection between RT and ferroptosis is the production of ROS. IR generates ROS directly through water radiolysis and indirectly through disruption of mitochondrial and enzymatic redox processes [69]. These ROS include hydroxyl radicals, superoxide anions, and hydrogen peroxide, which can damage DNA, proteins, and lipids. In the context of ferroptosis, the critical consequence is oxidation of PUFA-containing phospholipids within cellular membranes, because these lipids provide the substrate for chain-propagating lipid peroxidation reactions [6,46].

This link has been supported by experimental observations across different tumor models. Irradiated cancer cells display increased lipid ROS, malondialdehyde accumulation, and morphological changes consistent with ferroptosis, including condensed mitochondria with increased membrane density [63]. More importantly, functional interference with ferroptosis alters RT response, indicating that radiation-induced lipid oxidation is not simply a byproduct of oxidative injury but can contribute directly to cell killing [74]. In NPC, high-dose IR increased ROS, malondialdehyde, and Fe^2+^ levels while reducing ATP and GSH, together with upregulation of ACSL4, PTGS2, and IREB2 and downregulation of GPX4, supporting a dose-dependent induction of ferroptotic stress after irradiation [36]. Similarly, in irradiated HNSCC cells, RT-related experimental validation showed increased VDAC1 and TFRC and reduced GPX4, SLC7A11, and FTH1, again consistent with a pro-ferroptotic shift following irradiation [24].

Thus, one of the fundamental mechanisms by which RT induces ferroptosis is by creating an oxidative environment that initiates and amplifies membrane lipid peroxidation. Whether this oxidative burden culminates in ferroptotic death depends largely on the status of cellular defense systems and membrane lipid composition [4].

### 3.2. Suppression of the SLC7A11–GSH–GPX4 Axis After Irradiation

A second major mechanism is the disruption of the canonical anti-ferroptotic defense axis centered on SLC7A11, GSH, and GPX4. The system Xc^−^ transporter imports cystine for intracellular GSH synthesis, and GPX4 uses GSH to detoxify phospholipid hydroperoxides. When this axis is compromised, lipid peroxides accumulate and ferroptosis becomes more likely [75,76].

Several lines of evidence indicate that RT can weaken this pathway. First, irradiation directly consumes reducing equivalents and lowers intracellular antioxidant capacity, which diminishes available GSH [77]. Second, RT-associated oxidative stress can reduce GPX4 activity or expression [78]. Third, IR can suppress SLC7A11 expression, thereby limiting cystine uptake and further impairing GSH synthesis [79]. This mechanism is highly relevant because many radioresistant tumors appear to mount an adaptive increase in SLC7A11 and GPX4 after irradiation, effectively buffering radiation-induced lipid peroxidation and escaping ferroptosis [63].

HNC-related studies provide especially clear examples of this principle. In NPC, TXNIP overexpression enhanced radiosensitivity by increasing ROS and Fe^2+^, reducing xCT and GPX4 expression, lowering GSH, and promoting ferroptosis through the xCT-GSH-GPX4-ROS axis [30]. In another NPC study, CD38 promoted radioresistance by competitively binding TRIM21 and stabilizing SLC7A11 protein, thereby maintaining the SLC7A11/GSH/GPX4 axis and suppressing ferroptosis [27]. These data reinforce a broader concept already emphasized in pan-cancer RT–ferroptosis reviews: radiation may initially drive cells toward ferroptosis, but tumor cells can adapt by reinforcing the SLC7A11–GPX4 shield, making this axis a central determinant of radiosensitivity or radioresistance [5,10].

### 3.3. ATM-Dependent Signaling Links DNA Damage to Ferroptosis

One of the most important mechanistic bridges between classical radiobiology and ferroptosis is the ataxia–telangiectasia-mutated (ATM) pathway. ATM is activated by DNA double-strand breaks and orchestrates the canonical DNA damage response after irradiation [80]. However, emerging evidence suggests that ATM also contributes to RT-induced ferroptosis, at least in part through repression of SLC7A11 and associated metabolic consequences [79].

This is conceptually important because it connects radiation-induced genomic injury with lipid-based regulated cell death. Rather than operating as separate outcomes, DNA damage signaling and ferroptotic signaling may converge. In preclinical models, inhibition of ATM attenuated lipid oxidation and reduced RT-induced ferroptosis, indicating that ATM can function upstream of ferroptotic susceptibility after irradiation [81]. In this framework, RT-induced DNA damage activates ATM, ATM suppresses SLC7A11-dependent cystine uptake, intracellular GSH falls, and lipid peroxide detoxification weakens, thereby enabling ferroptosis [82].

This mechanism also offers a useful explanation for why some irradiated tumors undergo substantial non-apoptotic death despite relative apoptotic resistance. In cancers that retain a lipid environment favorable to peroxidation and have insufficient compensatory antioxidant adaptation, ATM signaling may help redirect the radiation response toward ferroptotic collapse rather than recovery.

### 3.4. ACSL4 Induction and Lipid Substrate Remodeling After Irradiation

RT-induced ferroptosis also depends on the availability of appropriate lipid substrates. Even under conditions of elevated ROS, ferroptosis cannot proceed efficiently unless membrane phospholipids contain oxidation-prone PUFAs [83]. ACSL4 is central to this process because it promotes formation of PUFA-containing phospholipids that serve as substrates for lipid peroxidation [50].

Multiple studies summarized in the uploaded RT–ferroptosis reviews indicate that irradiation increases ACSL4 expression and thereby enhances susceptibility to ferroptosis [84]. Conversely, ACSL4 deficiency reduces radiation-induced lipid peroxidation and confers radioresistance, demonstrating that ACSL4 is not merely a marker but a functional mediator of RT-associated ferroptotic death [85]. This concept is strongly supported by NPC data showing that high-dose irradiation upregulated ACSL4 together with other pro-ferroptotic genes [36]. In another NPC model, HAT1/HDAC2-mediated acetylation of ACSL4 increased radiosensitivity by promoting ferroptosis, further underscoring that post-translational control of ACSL4 can materially influence RT response [33].

These findings suggest that RT-induced ferroptosis is shaped not only by oxidant production but also by lipid remodeling. Tumors enriched in PUFA-phospholipids or driven toward ACSL4 activity are more likely to convert oxidative stress into lethal membrane damage. By contrast, tumors that shift toward MUFA-rich membranes can blunt ferroptosis and become more radioresistant [86].

### 3.5. Iron Metabolism as a Permissive Factor for Radiation-Induced Ferroptosis

Ferroptosis requires catalytically active iron, and RT appears capable of perturbing iron handling in ways that support ferroptotic injury. Iron participates in Fenton chemistry and accelerates lipid radical generation, thereby amplifying membrane peroxidation once oxidative stress is initiated [47]. Although the precise effects of RT on tumor iron metabolism may vary by context, increasing evidence indicates that changes in iron uptake, storage, and labile iron pools influence the ferroptotic consequences of irradiation [87].

In HNSCC, glutamine blockade combined with RT enhanced IRF1 expression, increased transferrin receptor expression, elevated intracellular Fe^2+^, and triggered immunogenic tumor ferroptosis, indicating that iron accumulation can be functionally coupled to radiation response [19]. In NPC patients and cells, post-RT changes in systemic iron-related indices together with elevated intracellular Fe^2+^ after high-dose irradiation further support the idea that iron metabolism is altered during RT and may contribute to ferroptosis induction [36]. These studies fit well with the broader model in which IR-induced ROS, together with increased iron availability, push tumor cells toward irreversible lipid peroxidation.

### 3.6. Adaptive Resistance: Why RT-Induced Ferroptosis Is Often Incomplete

Despite the pro-ferroptotic effects of irradiation, ferroptosis after RT is frequently partial, heterogeneous, or transient. One major reason is that tumor cells activate compensatory antioxidant and metabolic programs that limit lipid peroxidation and restore redox homeostasis. Among these adaptive mechanisms, NRF2 signaling is particularly important. Upon oxidative stress, NRF2 induces a broad antioxidant transcriptional program that includes SLC7A11 and other redox-protective genes, thereby antagonizing ferroptosis and promoting radioresistance [88,89].

Other adaptive responses include restoration of GPX4 or SLC7A11 expression after the initial radiation insult, increased MUFA biosynthesis through SREBP1/SCD1-related pathways, altered glutamine metabolism, and activation of parallel antioxidant systems such as FSP1-CoQ_10_ or BH_4_-related defenses [30,90,91,92]. HNC studies illustrate these adaptations clearly. In HNSCC, DDR1 sustained resistance to carbon ion radiotherapy by activating an Akt/mTORC1/SREBP1/SCD1 signaling cascade that promoted MUFA biosynthesis and suppressed ferroptosis; DDR1 inhibition disrupted this protective program and restored ferroptotic sensitivity [22]. In anaplastic thyroid carcinoma, YTHDF2-mediated stabilization of SREBF1 increased lipogenic enzyme expression including SCD1, suppressed ferroptosis, and promoted RT resistance through lipid metabolic remodeling [41]. In NPC, CD38 stabilized SLC7A11, while low TXNIP expression preserved antioxidant defenses and favored radioresistance [27,30].

Heat shock protein 27 (Hsp27/HSPB1) may represent another stress-adaptive mechanism linking radioresistance and ferroptosis suppression [93]. Hsp27 has been reported to protect tumor cells from radiation-induced oxidative injury by maintaining intracellular GSH levels and preserving the reduced redox state, thereby limiting ROS-mediated damage. In addition, HSPB1 negatively regulates ferroptotic cancer cell death by reducing iron-dependent lipid peroxidation and ferroptosis-associated oxidative stress [94]. Therefore, inhibition of Hsp27/HSPB1 may enhance post-radiation ferroptosis and represents a potential molecular strategy for radiosensitizing tumor cells, although its therapeutic relevance requires further validation in HNC-specific RT models.

Taken together, these observations indicate that RT-induced ferroptosis is opposed by dynamic cellular adaptation. This adaptive resistance likely explains why ferroptosis inducers or metabolic interventions often produce the greatest benefit when combined with RT rather than used alone.

### 3.7. Interactions Between RT-Induced Ferroptosis and Tumor Immunity

Recent work has extended the role of ferroptosis beyond direct tumor cell killing to include immune modulation. RT can activate inflammatory signaling, enhance antigen release, and alter the tumor microenvironment, while ferroptosis can influence immune cell recruitment, antigenicity, and phagocytic responses [95]. Accordingly, an emerging concept is that RT-induced ferroptosis may not only kill tumor cells directly but also reshape antitumor immunity [9].

This is particularly relevant in HNC. In HNSCC, glutamine inhibition combined with RT induced immunogenic tumor ferroptosis through an IRF1-TFRC-iron axis, but also increased CD47 expression, which hindered macrophage phagocytosis; combining glutamine blockade with CD47 inhibition improved tumor remission and enhanced RT-associated ferroptosis while favorably remodeling the microenvironment [19]. In carbon ion-irradiated HNSCC, DDR1 inhibition promoted ferroptosis-mediated immunogenic cell death and increased CD8^+^ T cell infiltration and cytotoxicity, suggesting that ferroptosis can amplify the immune consequences of RT when tumor lipid defenses are disabled [22]. These findings align with broader reviews proposing that RT, ATM signaling, and CD8^+^ T cell activity may cooperate to suppress SLC7A11, intensify lipid oxidation, and augment ferroptosis in tumors [79]. Thus, RT-induced ferroptosis should be considered not only a cell-autonomous death process but also part of a larger radiobiological network involving metabolic stress, danger signaling, and tumor–immune interaction.

### 3.8. Conceptual Synthesis

Overall, RT induces ferroptosis through a multi-layered process rather than a single pathway. IR first generates oxidative stress and membrane lipid damage; this is reinforced by depletion of GSH, impairment of GPX4 function, ATM-dependent repression of SLC7A11, induction of ACSL4 and PUFA-phospholipid synthesis, and changes in iron metabolism. The ultimate outcome is then shaped by adaptive resistance programs such as NRF2 activation, SLC7A11/GPX4 rebound, and MUFA-centered lipid remodeling. If pro-ferroptotic pressure predominates, irradiated cells undergo ferroptotic death; if antioxidant and lipid-protective adaptation prevails, radioresistance emerges [10,63].

This framework is highly relevant to HNC, where recent original studies increasingly show that RT response is closely linked to ferroptosis-regulatory pathways, including xCT-GSH-GPX4 signaling, ACSL4 activation, iron homeostasis, lipid desaturation, and immune crosstalk. These disease-specific data provide a strong rationale for the next section, which focuses on ferroptosis as a determinant of radiosensitivity and radioresistance in head and neck malignancies.

## 4. Ferroptosis as a Determinant of Radiosensitivity and Radioresistance in Head and Neck Cancer

**Figure 2:**
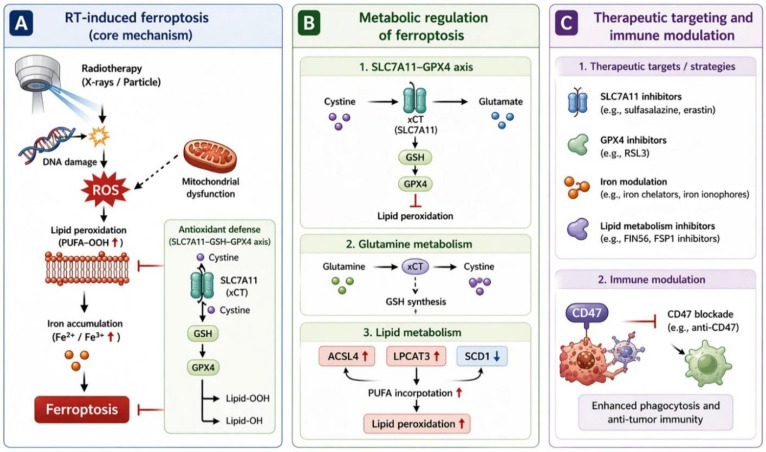
Ferroptosis-mediated radiosensitization pathways in head and neck cancer. RT promotes ferroptosis through interconnected mechanisms involving oxidative stress, metabolic regulation, and therapeutic targeting. (**A**) Ionizing radiation induces DNA damage and reactive oxygen species (ROS), leading to lipid peroxidation and iron accumulation that drive ferroptosis. (**B**) Ferroptosis sensitivity is regulated by metabolic pathways, including the SLC7A11–GSH–GPX4 antioxidant axis, glutamine metabolism, and lipid remodeling via ACSL4/LPCAT3 (increased) and SCD1 (decreased). (**C**) Therapeutic strategies include targeting antioxidant defenses, iron metabolism, and lipid pathways, as well as immune modulation such as CD47 blockade, which enhances macrophage-mediated clearance of ferroptotic tumor cells. Abbreviations: ACSL4, acyl-CoA synthetase long-chain family member 4; GSH, glutathione; GPX4, glutathione peroxidase 4; LPCAT3, lysophosphatidylcholine acyltransferase 3; PUFA, polyunsaturated fatty acid; ROS, reactive oxygen species; RT, radiotherapy; SCD1, stearoyl-CoA desaturase 1; SLC7A11 (xCT), cystine/glutamate antiporter system component.

In HNC, the biological outcome of RT is shaped not only by the extent of DNA damage but also by how efficiently irradiated tumor cells undergo oxidative membrane injury and ferroptotic collapse. This concept is particularly relevant because head and neck malignancies are frequently treated with definitive or adjuvant RT, yet local failure and recurrent disease remain closely linked to intrinsic or acquired radioresistance [96]. Across the currently available studies, a recurring theme is that radiosensitive tumors tend to permit radiation-driven lipid peroxidation, iron dysregulation, and antioxidant failure, whereas radioresistant tumors preserve redox homeostasis through the reinforcement of anti-ferroptotic pathways, such as SLC7A11–GSH–GPX4 signaling, MUFA synthesis, and metabolic buffering (Table 1, Figure 2). Importantly, ferroptosis-related mechanisms are not uniform across head and neck malignancies. Distinct tumor entities—including HPV-positive and HPV-negative HNSCC, NPC, OSCC, and thyroid cancers—exhibit different radiotherapy sensitivities, metabolic adaptations, and ferroptosis regulatory profiles. To better delineate these differences, a comparative overview of tumor-specific ferroptosis characteristics is provided in Table 2. In this framework, ferroptosis is not merely one of many downstream events after irradiation, but an important biological discriminator between effective tumor control and therapeutic escape [30,90,91,92].

### 4.1. Head and Neck Squamous Cell Carcinoma: Ferroptosis Competence as a Radiosensitivity Trait

The HNSCC data increasingly support the idea that the capacity to engage ferroptosis is itself a determinant of radiation response. In a clinically oriented study of HPV-negative HNSCC, Noh et al. identified a ferroptosis-associated gene signature linked to radiotherapy response and showed that statin treatment enhanced lipid peroxidation and improved RT efficacy, suggesting that ferroptosis-prone tumors may be selectively radiosensitized through metabolic intervention [20]. Similarly, Reinema et al. demonstrated that MitoTam increased ROS, disrupted mitochondrial membrane potential, induced lipid peroxidation, and sensitized both radiosensitive and radioresistant HNSCC cells to RT in a ferroptosis-dependent manner, indicating that elevated antioxidant buffering in resistant cells can be therapeutically overcome by forcing oxidative membrane damage [21]. These findings fit well with the broader concept that HNSCC radiosensitivity depends partly on whether RT-induced ROS can be translated into lethal lipid oxidation rather than neutralized by tumor antioxidant reserves.

Additional support comes from studies that link ferroptosis-associated markers to prognosis and treatment stratification. Cao et al. developed a prognostic signature indicating that ferroptosis-related biology is associated with radiosensitivity in HNSCC and further showed that MTDH enhanced radiosensitivity by promoting ferroptosis [23]. Fan et al. later identified ferroptosis- and RT-related genes with diagnostic and prognostic significance and experimentally observed RT-associated upregulation of VDAC1 and TFRC together with downregulation of GPX4, SLC7A11, and FTH1 in irradiated HNSCC cells [24]. Although these signature-based studies do not establish causality as directly as intervention experiments, they are important because they suggest that ferroptosis status is measurable at the molecular level and may be clinically informative for predicting treatment response, immune context, and individualized radiosensitization strategies.

Song et al. provided particularly strong mechanistic evidence that ferroptosis is functionally linked to radiosensitivity in HNSCC [19]. They showed that after RT, glutamine levels and SLC1A5 expression increased in HNSCC, whereas glutamine blockade enhanced RT efficacy by inducing immunogenic tumor ferroptosis. Mechanistically, RT activated IRF1, and glutamine inhibition amplified this response, leading to transferrin receptor upregulation, intracellular Fe^2+^ accumulation, and ferroptotic cell death. Importantly, the benefit of glutamine blockade was attenuated by compensatory CD47 upregulation, while concurrent CD47 inhibition improved tumor remission and microenvironmental remodeling. This study is notable because it places ferroptosis at the intersection of metabolism, iron homeostasis, RT, and immune evasion rather than treating it as an isolated death program [19].

A related but distinct line of evidence comes from carbon ion RT. Hu et al. showed that DDR1 promoted resistance to carbon ion irradiation in HNSCC by sustaining an Akt/mTORC1/SREBP1/SCD1 signaling axis that favored monounsaturated fatty acid biosynthesis and suppressed ferroptosis [22]. Genetic or pharmacologic inhibition of DDR1 disrupted this lipid-protective program, enhanced ferroptosis-mediated immunogenic cell death, increased CD8^+^ T-cell infiltration, and improved tumor control. This study is important because it highlights that radioresistance in HNSCC can be actively maintained by lipid remodeling, especially through SREBP1/SCD1-mediated membrane desaturation, and that disabling this adaptation can restore ferroptotic susceptibility even in the context of advanced particle therapy.

Taken together, the HNSCC literature suggests that radiosensitivity is favored when tumor cells fail to defend against RT-triggered iron-dependent lipid peroxidation, whereas radioresistance emerges when metabolic and antioxidant adaptations preserve membrane integrity. This principle appears to apply across conventional photon RT and carbon ion RT and is supported by interventional, molecular-signature, and immune-mechanistic studies.

### 4.2. Nasopharyngeal Carcinoma: Ferroptosis Suppression as a Central Mechanism of Radioresistance

Among head and neck malignancies, NPC currently has the richest body of mechanistic evidence linking ferroptosis to radioresponse [99]. Several studies indicate that irradiation itself can induce ferroptotic stress in NPC, but radioresistant cells adapt by strengthening antioxidant pathways, altering lipid handling, and stabilizing ferroptosis suppressors. This makes NPC an especially useful model for understanding how ferroptosis competence is gained or lost during RT.

Direct evidence that irradiation can trigger ferroptosis in NPC has accumulated steadily. Yang et al. showed that ionizing radiation upregulated ferroptosis-related genes, increased ROS, disrupted mitochondrial membrane potential, altered GSH levels, and elevated lipid peroxidation markers, while SLC7A11 depletion further sensitized NPC cells to IR [25]. Chen et al. similarly found that IR promoted lipid peroxidation and ferroptosis in NPC cells and that GSTM3 enhanced radiosensitivity by promoting this response through the USP14/FASN axis and GPX4 [32]. More recently, Jin et al. demonstrated that high-dose IR increased ACSL4, PTGS2, and IREB2 expression, decreased GPX4, elevated ROS, malondialdehyde, and Fe^2+^, reduced ATP and GSH, and induced G2/M arrest, supporting a dose-dependent ferroptotic contribution to RT response in NPC [36]. Together, these studies indicate that NPC cells can indeed undergo irradiation-induced ferroptosis, but the extent of this response varies with dose, cellular context, and anti-ferroptotic adaptation.

A major determinant of NPC radioresistance is preservation of the SLC7A11–GSH–GPX4 axis. Zhou et al. showed that TXNIP expression was lower in radioresistant NPC and that TXNIP overexpression increased ROS, reduced xCT and GPX4, altered Fe^2+^, MDA, and GSH, and restored radiosensitivity through the xCT-GSH-GPX4-ROS axis [30]. Li et al. further demonstrated that CD38 promoted RT resistance by competitively binding TRIM21 and stabilizing SLC7A11 protein, thereby sustaining the SLC7A11/GSH/GPX4 pathway and suppressing ferroptosis [27]. Dai et al. added an epitranscriptomic dimension by showing that METTL3-mediated m^6^A modification of SLC7A11 enhanced NPC radioresistance through ferroptosis inhibition [26]. Collectively, these studies converge on the same central principle: persistent maintenance of cystine import, GSH metabolism, and GPX4-mediated peroxide detoxification is a core mechanism by which NPC escapes ferroptotic death after irradiation.

NPC radioresistance is also shaped by broader metabolic and lipid-remodeling programs. Huang et al. showed that local angiotensin II signaling promoted radioresistance through a HIF-1α-HILPDA axis that enhanced lipid droplet accumulation and suppressed ferroptosis, linking hypoxia-related signaling to protection from lipid peroxidation [28]. Mi et al. found that lncRNA HOTAIRM1 promoted radioresistance by modulating FTO acetylation-dependent alternative splicing of CD44 and attenuating irradiation-induced ferroptosis [29]. He et al. reported that downregulation of PCK2 enhanced the radioresistant phenotype of NPC, with the data suggesting that loss of PCK2 weakened radiation-induced ferroptosis [31]. Xu et al. described RRFERV as a lncRNA associated with radiation resistance and poor outcome; however, they also observed that ferroptosis induction rendered these tumor cells vulnerable, implying that ferroptosis-targeted intervention may overcome an otherwise aggressive resistant state [97]. These studies emphasize that in NPC, ferroptosis suppression is not controlled by one pathway alone; instead, radioresistance arises from integrated regulation of antioxidant defense, metabolic flux, lipid storage, and gene-expression circuitry.

Several studies have also shown that deliberate promotion of ferroptosis can reverse radioresistance in NPC. Amos et al. demonstrated that SOD2 depletion increased oxidative stress, lipid peroxidation, and radiosensitivity via ferroptosis induction, while also showing that this effect depended on DHODH activity, an observation that is important when considering mitochondrial redox-targeted combinations [35]. Zhou et al. showed that HAT1/HDAC2-mediated ACSL4 acetylation conferred radiosensitivity by inducing ferroptosis in NPC [33]. Wang et al. used nanocarriers targeting circADARB1 to boost radiosensitivity through synergistic promotion of ferroptosis [34]. Itraconazole was also reported to attenuate NPC stemness by triggering ferroptosis, suggesting that ferroptosis induction may help counter stem-like treatment-resistant phenotypes relevant to recurrence after RT [98]. Viewed together, these studies strongly support ferroptosis induction as an actionable radiosensitization strategy in NPC, with interventions ranging from antioxidant disruption and lipid remodeling to RNA-targeted and nanomedicine-based approaches.

Overall, the NPC evidence suggests that radioresistance reflects an actively maintained anti-ferroptotic state. Radiosensitivity improves when this state is dismantled, whether by reducing SLC7A11/GPX4 protection, increasing ACSL4-driven lipid vulnerability, enhancing oxidative stress, or blocking hypoxia- and lipid droplet-associated buffering.

### 4.3. Oral Squamous Cell Carcinoma: Ferroptosis-Mediated Re-Sensitization of Resistant Cells

Although the OSCC literature is less extensive than that of HNSCC or NPC, the available studies still support a functional role for ferroptosis in modulating RT response. Liu et al. showed that hyperbaric oxygen enhanced X-ray-induced ferroptosis and re-sensitized radioresistant OSCC cells through GPX4/ferroptosis regulation [37]. Du et al. reported that astaxanthin synergized with IR in OSCC and implicated ferroptosis-related mechanisms, including GPX4-associated regulation [38]. Cheng et al. observed that cold atmospheric plasma jet irradiation reduced the survival of OSCC cells and altered oncogenic miRNA expression, with the pattern of oxidative injury and death signaling suggesting potential overlap with ferroptosis-related processes [39]. While these studies vary in mechanistic depth, they consistently indicate that the radiosensitivity of oral cancer cells can be enhanced by interventions that intensify oxidative membrane stress or weaken ferroptosis defenses. Beyond individual experimental studies, recent comprehensive analyses indicate that OSCC exhibits intrinsic susceptibility to ferroptosis induction, even in treatment-resistant contexts, and that ferroptosis inducers can exert significant anti-tumor effects [73]. Moreover, ferroptosis has been proposed as an immunogenic form of cell death in OSCC, potentially contributing to modulation of anti-tumor immunity and therapeutic response.

### 4.4. Thyroid Carcinoma and Anaplastic Thyroid Carcinoma: Glutathione Depletion and Lipid Desaturation Define the Response

The thyroid cancer studies in the dataset further reinforce the general principle that radiosensitivity is promoted by ferroptosis induction, whereas radioresistance is maintained by metabolic adaptation. In thyroid carcinoma, Yang et al. showed that CHAC1 promoted ferroptosis and enhanced radiation sensitivity, consistent with the established role of CHAC1 in GSH degradation and redox imbalance [40]. This is mechanistically coherent with the broader view that weakening GSH-dependent peroxide detoxification lowers the threshold for ferroptosis after irradiation.

In contrast, Dai et al. demonstrated in anaplastic thyroid carcinoma that YTHDF2-mediated stabilization of SREBF1 enhanced expression of downstream lipogenic enzymes, including SCD1, remodeled membrane lipids, suppressed ferroptosis, and promoted radioresistance [41]. Reintroduction of SREBF1 restored radiotolerance and reversed ferroptotic susceptibility in YTHDF2-deficient cells, while inhibition of this pathway sensitized tumors to ionizing radiation in vivo. These findings are highly informative because they mirror the HNSCC DDR1/SREBP1/SCD1 story and suggest that monounsaturated lipid remodeling is a recurrent anti-ferroptotic mechanism across distinct head and neck malignancies.

### 4.5. Conceptual Implications for Head and Neck Radiobiology

A common pattern emerges across HNSCC, NPC, OSCC, and thyroid malignancies. Radiosensitivity is associated with increased iron-dependent lipid peroxidation, suppression of the xCT/SLC7A11–GSH–GPX4 axis, ACSL4 activation, GSH depletion, or failure of lipid-protective remodeling. Radioresistance, by contrast, is associated with stabilization of SLC7A11, restoration of GPX4 function, maintenance of GSH pools, hypoxia-related lipid droplet accumulation, enhanced MUFA biosynthesis through SREBP1/SCD1, or broader metabolic rewiring that neutralizes radiation-induced oxidative stress. In other words, head and neck tumors appear to differ not only in how much ROS they generate after RT, but in whether they can convert that stress into lethal ferroptotic membrane injury.

This interpretation also has translational implications. First, ferroptosis-related molecules such as SLC7A11, GPX4, ACSL4, TFRC, SCD1, and pathway-level signatures may serve as biomarkers of radioresponse. Second, radiosensitization strategies in HNC may need to be tailored according to the dominant mode of ferroptosis suppression in a given tumor, such as antioxidant reinforcement, lipid remodeling, mitochondrial buffering, or immune evasion. Third, because several studies now connect ferroptosis to immunogenic cell death and microenvironmental change, the biological effect of ferroptosis extends beyond direct tumor cell killing and may influence how RT interfaces with immunotherapy or macrophage-mediated clearance. The cumulative evidence therefore supports ferroptosis as both a mechanistic determinant and a therapeutically actionable vulnerability in head and neck RT.

## 5. Therapeutic Strategies to Induce Ferroptosis for Head and Neck Cancer Radiotherapy

**Figure 3:**
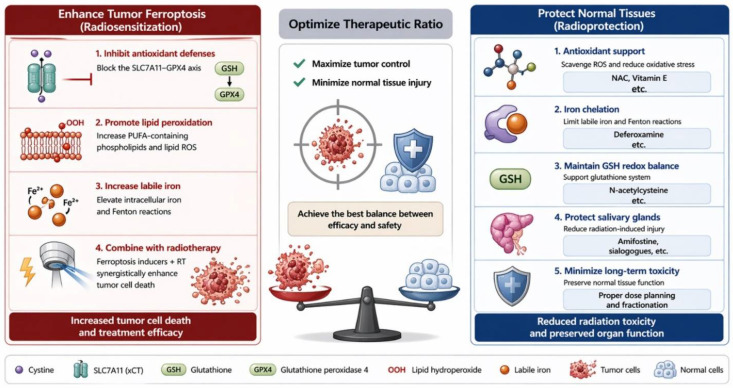
Therapeutic balance between tumor ferroptosis induction and normal tissue protection in head and neck cancer radiotherapy. Enhancing ferroptosis in tumor cells can improve radiosensitivity through inhibition of antioxidant defenses, promotion of lipid peroxidation, and increased intracellular iron. In contrast, normal tissue protection requires suppression of excessive oxidative damage via antioxidant support, iron regulation, and maintenance of redox balance. Achieving an optimal therapeutic ratio depends on selectively inducing ferroptosis in tumor cells while minimizing toxicity in surrounding normal tissues, particularly the salivary glands and oral mucosa. Abbreviations: GPX4, glutathione peroxidase 4; GSH, glutathione; ROS, reactive oxygen species; RT, radiotherapy; SLC7A11 (xCT), cystine/glutamate antiporter system component; PUFA, polyunsaturated fatty acid.

The emerging recognition that ferroptosis contributes to radiation-induced tumor cell death has stimulated growing interest in therapeutic strategies designed to deliberately promote ferroptosis during RT. In HNC, such strategies are particularly attractive because RT remains a cornerstone of treatment for both definitive and adjuvant settings, yet intrinsic and acquired radioresistance frequently limit local control. Current experimental evidence suggests that ferroptosis induction can enhance radiosensitivity through several complementary mechanisms, including disruption of antioxidant defenses, promotion of lipid peroxidation, metabolic reprogramming, modulation of iron metabolism, and immune-mediated tumor clearance (Table 1). These approaches are beginning to reveal practical pathways through which ferroptosis may be exploited to improve the therapeutic efficacy of RT in head and neck malignancies (Figure 3).

### 5.1. Targeting the SLC7A11–GSH–GPX4 Axis to Enhance Radiosensitivity

The most direct approach to promoting ferroptosis during RT is inhibiting cellular antioxidant systems that normally suppress lipid peroxidation. Among these, the SLC7A11–GSH–GPX4 axis plays a central role in protecting tumor cells from ferroptotic damage by maintaining cystine uptake, GSH synthesis, and lipid peroxide detoxification. Several studies in HNC models indicate that disruption of this pathway can markedly enhance the cytotoxic effects of RT.

In NPC, suppression of SLC7A11 has been shown to increase radiosensitivity by amplifying lipid peroxidation and ferroptotic stress following irradiation [25]. Conversely, stabilization of the SLC7A11 protein can promote radioresistance. For example, CD38 has been reported to enhance RT resistance in NPC by competitively binding TRIM21 and preventing SLC7A11 degradation, thereby sustaining the SLC7A11/GSH/GPX4 antioxidant axis and suppressing ferroptosis [27]. Epitranscriptomic regulation also contributes to this process; METTL3-mediated m^6^A modification of SLC7A11 transcripts has been shown to reinforce ferroptosis resistance and diminish radiosensitivity [26].

Additional evidence highlights the role of redox regulators in modulating this pathway. In radioresistant nasopharyngeal carcinoma cells, reduced expression of TXNIP preserves the xCT-GSH-GPX4 system and attenuates ferroptosis, whereas TXNIP overexpression promotes lipid peroxidation, increases intracellular Fe^2+^, and restores radiosensitivity [30]. Similarly, GSTM3 has been shown to enhance radiosensitivity by promoting ferroptosis through modulation of the USP14/FASN/GPX4 signaling axis [32]. Collectively, these findings indicate that weakening the antioxidant shield centered on SLC7A11 and GPX4 represents one of the most promising strategies for inducing ferroptosis in irradiated head and neck tumors.

### 5.2. Manipulating Lipid Metabolism to Increase Susceptibility to Ferroptosis

Ferroptosis depends critically on the availability of oxidizable polyunsaturated phospholipids within cellular membranes. Consequently, therapeutic strategies that modify lipid metabolism to favor peroxidation-prone phospholipid composition may enhance RT efficacy. Several studies highlight the importance of lipid remodeling in determining radiation response. In NPC, acetylation of ACSL4 has been shown to increase ferroptosis and enhance radiosensitivity, demonstrating that post-translational regulation of lipid metabolic enzymes can influence RT response [33]. Conversely, tumor cells can escape ferroptosis through lipid desaturation programs that enrich membranes with MUFA. In head and neck squamous cell carcinoma, DDR1 has been shown to maintain resistance to carbon ion radiotherapy through activation of an Akt/mTORC1/SREBP1/SCD1 signaling pathway that promotes MUFA synthesis and suppresses ferroptosis [22]. Inhibition of DDR1 disrupts this lipid remodeling program, increases ferroptotic cell death, and enhances the efficacy of carbon ion irradiation.

A similar mechanism has been observed in anaplastic thyroid carcinoma, where YTHDF2-mediated stabilization of SREBF1 enhances SCD1 expression, alters membrane lipid composition, suppresses ferroptosis, and promotes radioresistance [41]. Disruption of this pathway restores ferroptotic susceptibility and improves radiation response in vivo. Together, these studies underscore the central importance of lipid metabolic state in determining whether radiation-induced oxidative stress leads to ferroptotic death or adaptive survival.

### 5.3. Targeting Tumor Metabolism and Iron Homeostasis

Metabolic interventions that influence iron availability or oxidative stress can also enhance ferroptosis during RT. Tumor cells often reprogram nutrient metabolism to maintain redox balance, and interfering with these pathways can shift the balance toward ferroptotic vulnerability. In HNSCC, glutamine metabolism has been shown to play a key role in regulating ferroptosis during RT. Glutamine blockade increases IRF1 expression following irradiation, elevates transferrin receptor levels, promotes intracellular Fe^2+^ accumulation, and induces immunogenic ferroptosis in tumor cells [19]. Importantly, this effect can be amplified by inhibiting CD47, which enhances macrophage-mediated clearance of ferroptotic tumor cells and improves tumor control.

Other metabolic regulators have also been implicated in ferroptosis-associated radiosensitivity. In thyroid carcinoma, CHAC1 promotes GSH degradation and enhances radiation sensitivity by promoting ferroptosis [40]. In NPC, downregulation of PCK2 has been associated with impaired ferroptotic responses and enhanced radioresistance, further highlighting the importance of metabolic state in determining ferroptotic competence [31]. These findings collectively suggest that metabolic vulnerabilities—including glutamine metabolism, iron handling, and GSH turnover—can be leveraged to promote ferroptosis and improve RT outcomes.

### 5.4. Nanotechnology and RNA-Targeted Approaches to Promote Ferroptosis

In addition to metabolic and pharmacologic interventions, emerging nanotechnology-based strategies are being developed to enhance ferroptosis during RT. These approaches are designed to deliver ferroptosis-inducing agents or genetic regulators directly to tumor cells, thereby increasing therapeutic specificity while minimizing systemic toxicity.

One example is the use of nanocarriers targeting circADARB1 in NPC [34]. By silencing this circular RNA, investigators demonstrated enhanced ferroptosis and increased radiosensitivity in tumor cells, suggesting that RNA-targeted nanomedicine may provide a promising strategy for overcoming RT resistance. Other studies have shown that SOD2 depletion can increase lipid peroxidation and radiosensitivity via ferroptosis induction, highlighting additional opportunities for redox-targeted interventions [35].

Nanotechnology-based delivery systems may also facilitate combination strategies in which ferroptosis-inducing drugs are co-administered with RT [34]. Because ferroptosis depends on local lipid oxidation and iron-dependent radical propagation, targeted delivery of these agents to tumor tissues could potentially enhance tumor-specific radiosensitization while minimizing normal tissue toxicity.

### 5.5. Therapeutic Implications

Collectively, the available evidence indicates that ferroptosis induction represents a promising strategy to enhance radiotherapy in HNC. Approaches targeting antioxidant defenses, lipid metabolism, metabolic vulnerabilities, and tumor microenvironmental interactions have all demonstrated the ability to increase radiation-induced ferroptosis in preclinical models. Importantly, several of these mechanisms—such as inhibition of SLC7A11, modulation of ACSL4 activity, disruption of lipid desaturation pathways, and metabolic targeting of glutamine or iron metabolism—are increasingly recognized as central regulators of radioresponse across multiple head and neck malignancies.

Modulation of ferroptosis through systemic metabolic interventions, including dietary factors, has been proposed as a potential strategy to enhance therapeutic efficacy. For example, increasing the availability of PUFAs such as AA or redox-active molecules such as ascorbate could, in theory, augment lipid peroxidation and ferroptosis sensitivity. However, such approaches remain speculative and may carry risks, as systemic increases in iron or pro-oxidant substrates could also exacerbate oxidative damage in normal tissues exposed to RT. Therefore, further studies are required to determine the safety and feasibility of these strategies in clinical settings.

At the same time, the translational development of ferroptosis-based radiosensitization strategies must carefully consider tumor heterogeneity and treatment-associated toxicity. The balance between therapeutic ferroptosis induction in tumor cells and unintended ferroptotic injury in surrounding normal tissues will ultimately determine the clinical feasibility of these approaches. Understanding this balance will therefore be essential for the safe integration of ferroptosis-targeted therapies into RT protocols for head and neck cancer. To further bridge mechanistic insights with clinical application, key translational considerations for ferroptosis-based radiosensitization are summarized below.

### 5.6. Translational Considerations for Ferroptosis-Based Radiosensitization

Despite promising preclinical evidence, several key translational questions remain to be addressed for the clinical implementation of ferroptosis-based radiosensitization strategies. First, only a limited number of ferroptosis-inducing approaches are currently druggable in clinical settings. Inhibition of the SLC7A11–GSH–GPX4 axis using agents such as sulfasalazine or targeting lipid peroxidation pathways represents a feasible strategy, whereas emerging targets such as FSP1 and DHODH remain largely preclinical. Second, the toxicity profile of ferroptosis induction must be carefully considered. Because ferroptosis is driven by oxidative lipid damage, excessive activation may exacerbate radiation-induced injury in normal tissues, particularly in the salivary glands and oral mucosa. Therefore, tumor-selective targeting strategies are essential to improve the therapeutic ratio. Third, integration with cisplatin-based chemoradiation requires further investigation. Cisplatin can enhance oxidative stress and GSH depletion, suggesting potential synergy with ferroptosis induction, but combined redox modulation may also increase systemic toxicity. Fourth, biomarker-driven patient selection will be critical for clinical translation. Expression of SLC7A11, GPX4, ACSL4, and iron-metabolism-related genes, as well as lipid composition and redox status, may help identify tumors that are more susceptible to ferroptosis. Fifth, the timing of ferroptosis induction relative to fractionated RT remains an unresolved issue. Because oxidative stress accumulates during repeated radiation exposure, ferroptosis-targeting strategies may be most effective when synchronized with peak oxidative burden. Finally, ferroptosis may interact with immunotherapy. As an immunogenic form of cell death, ferroptosis has the potential to enhance anti-tumor immune responses and may synergize with immune checkpoint blockade, including PD-1/PD-L1 inhibitors. However, excessive ferroptosis could also impair immune cell function, highlighting the need for balanced modulation.

## 6. Ferroptosis and Radiation-Induced Normal Tissue Injury in the Head and Neck Region

While ferroptosis induction has emerged as a promising strategy to enhance tumor radiosensitivity, its implications for surrounding normal tissues are more complex. This issue is particularly important in head and neck RT because treatment fields frequently encompass critical structures such as salivary glands, oral mucosa, pharyngeal constrictors, skin, and connective tissues that are essential for speech, swallowing, taste, and lubrication. Consequently, the same oxidative lipid damage that may improve tumor control can also contribute to normal tissue toxicity if ferroptosis is activated in healthy cells (Figure 3). Increasing evidence from radiobiology research indicates that ferroptosis may participate in both tumor suppression and radiation-induced tissue injury, highlighting its dual role in determining the therapeutic ratio of RT [5,7,8,10,100].

### 6.1. Ferroptosis as a Potential Mechanism of Radiation-Induced Tissue Injury

Radiation-induced normal tissue injury has traditionally been explained through DNA damage, oxidative stress, vascular dysfunction, inflammatory cytokine release, and progressive fibrosis. However, the biochemical environment generated by ionizing radiation—characterized by ROS accumulation, iron-dependent redox reactions, GSH depletion, and membrane lipid peroxidation—closely resembles the molecular conditions that drive ferroptosis. These shared features have led to the proposal that ferroptosis represents one of the regulated cell-death pathways contributing to radiation injury in normal tissues [74,84].

Experimental evidence from multiple organ systems supports this concept. Radiation exposure can increase lipid peroxidation, disrupt antioxidant defenses, and alter iron metabolism in normal cells, thereby promoting ferroptotic damage. In several experimental models, inhibition of ferroptosis has attenuated radiation-induced inflammation, tissue degeneration, or fibrosis, suggesting that lipid peroxide accumulation may contribute to the pathogenesis of radiation injury rather than merely reflecting secondary oxidative stress [101,102]. Although most mechanistic studies have focused on organs such as the lung, intestine, and hematopoietic system, the same biochemical principles are likely relevant to tissues exposed during head and neck RT.

### 6.2. Salivary Gland Dysfunction as a Clinically Relevant Example

Among normal tissues affected by head and neck RT, the salivary glands represent the most clinically significant example in which ferroptosis may contribute to treatment-related toxicity. Radiation-induced salivary gland dysfunction frequently leads to xerostomia, oral discomfort, impaired swallowing, dental deterioration, and reduced quality of life. Despite advances in intensity-modulated radiotherapy (IMRT) and other conformal techniques, salivary gland injury remains a common long-term complication in patients treated for HNC.

Recent work has proposed that ferroptosis may represent an additional mechanism underlying radiation-induced salivary gland damage [43,103]. In this context, oxidative stress and lipid peroxidation generated by irradiation may trigger ferroptotic injury in acinar cells and glandular progenitors, thereby impairing salivary secretion and tissue regeneration. Ferroptosis may also contribute to sustained inflammation and fibrotic remodeling, processes that further limit recovery of glandular function after treatment. Although direct experimental evidence in salivary gland tissue remains limited, the biological plausibility of this mechanism and the broader literature linking ferroptosis to radiation injury support its consideration in the pathogenesis of xerostomia and related complications.

### 6.3. Acute Versus Late Radiation Toxicity

Radiation-induced toxicity in the head and neck region can be broadly divided into acute and late phases, each of which may involve distinct biological mechanisms. Acute toxicity occurs during or shortly after treatment and includes mucositis, dermatitis, and early glandular dysfunction. Late toxicity may emerge months or years later and often involves fibrosis, vascular damage, and chronic tissue dysfunction.

Ferroptosis may contribute to both phases through different mechanisms [104,105]. During the acute phase, radiation-induced ROS production and GSH depletion may trigger ferroptotic death in epithelial or secretory cells, leading to early tissue injury [106]. In the chronic phase, persistent lipid peroxidation and inflammatory signaling associated with ferroptosis may contribute to long-term tissue remodeling and fibrotic processes [107]. In this way, ferroptosis may act as a biological bridge connecting early oxidative injury to late structural damage in irradiated tissues.

### 6.4. Implications for the Therapeutic Ratio of Radiotherapy

The dual role of ferroptosis in tumor control and normal tissue injury creates an important therapeutic challenge. In tumor cells, induction of ferroptosis may enhance radiosensitivity, overcome resistance, and improve treatment efficacy. In normal tissues, however, excessive ferroptosis may exacerbate radiation toxicity and compromise patient quality of life. The overall clinical impact, therefore, depends on whether ferroptosis can be selectively induced in tumors while minimizing damage to surrounding healthy tissues. Achieving this balance will likely require strategies that exploit tumor-specific vulnerabilities [82]. Many HNCs exhibit increased iron metabolism, altered lipid composition, and elevated oxidative stress compared with normal tissues. These features may create a therapeutic window in which ferroptosis-inducing interventions preferentially affect tumor cells while sparing normal structures. Advances in targeted drug delivery, metabolic modulation, and precision RT techniques may further help maintain this differential sensitivity [108].

### 6.5. Potential Radioprotective Strategies Targeting Ferroptosis

Understanding ferroptosis in normal tissues also raises the possibility of using ferroptosis inhibition as a radioprotective strategy. If lipid peroxidation contributes to radiation-induced damage in organs such as the salivary glands, targeted suppression of ferroptotic pathways in normal tissues might reduce toxicity without compromising tumor control. Preclinical studies in other organ systems have shown that inhibition of ferroptosis can mitigate radiation-induced tissue injury and inflammation, suggesting that similar approaches may eventually be explored in the head and neck setting [7,100]. Future research should therefore focus on identifying the specific cellular compartments in which ferroptosis occurs within irradiated head and neck tissues and determining whether these processes are causally responsible for long-term functional impairment. Such work will be critical for designing therapeutic strategies that enhance tumor-directed ferroptosis while preserving normal tissue integrity.

### 6.6. Summary

In summary, ferroptosis appears to function as a double-edged regulator of RT outcomes in the head and neck region. On one hand, ferroptosis induction offers an attractive mechanism for enhancing tumor radiosensitivity. On the other hand, uncontrolled ferroptotic damage in normal tissues may contribute to treatment-related toxicity. The challenge for future therapeutic development is therefore not simply to increase ferroptosis during RT, but to achieve tumor-selective ferroptosis while protecting surrounding organs. In HNC, where preservation of speech, swallowing, and salivary function is essential, this balance will likely determine the ultimate clinical value of ferroptosis-targeted radiosensitization strategies [20,21].

## 7. Clinical Implications, Current Limitations, and Future Directions

The growing body of experimental and translational research indicates that ferroptosis plays an important role in shaping the HNC response to RT. Across multiple tumor types—including HNSCC, NPC, OSCC, and thyroid malignancies—studies consistently demonstrate that modulation of ferroptosis can alter radiosensitivity, tumor growth control, and resistance to treatment. These findings collectively suggest that ferroptosis represents a biologically meaningful mechanism underlying RT response and a potentially actionable vulnerability that could be exploited to improve therapeutic outcomes in head and neck oncology.

### 7.1. Clinical Implications of Ferroptosis in Radiotherapy

One of the most important clinical implications of these findings is the possibility of developing ferroptosis-based radiosensitization strategies. Several molecular pathways that regulate ferroptosis have already emerged as promising therapeutic targets. Disruption of the SLC7A11–GSH–GPX4 antioxidant axis, enhancement of ACSL4-mediated lipid peroxidation, inhibition of lipid desaturation pathways such as SREBP1–SCD1 signaling, and manipulation of iron metabolism have all been shown to increase radiation-induced ferroptosis in preclinical models. These approaches have demonstrated the ability to overcome radioresistance in HNC experimental systems, suggesting that pharmacologic or genetic modulation of ferroptosis may provide a new class of radiosensitizing interventions.

Beyond direct tumor radiosensitization, ferroptosis may also influence the interaction between RT and the tumor microenvironment. Radiation-induced ferroptosis has been linked to immunogenic tumor cell death, increased antigen presentation, and enhanced recruitment of immune cells. In HNSCC, metabolic targeting of glutamine has been shown to induce immunogenic ferroptosis during RT and reshape the tumor microenvironment through iron-dependent oxidative stress and macrophage-mediated tumor clearance. These observations raise the possibility that ferroptosis induction may augment not only the cytotoxic effects of RT but also its immunomodulatory consequences, potentially improving responses to immunotherapy combinations [9,19].

### 7.2. Current Limitations and Challenges

Despite these promising developments, several challenges must be addressed before ferroptosis-targeted radiosensitization strategies can be translated into clinical practice. First, the heterogeneity of ferroptosis susceptibility among tumors remains incompletely understood. Different HNCs display distinct metabolic profiles, lipid compositions, and antioxidant capacities, all of which influence ferroptotic vulnerability. For example, stabilization of SLC7A11, metabolic reprogramming involving lipid desaturation pathways, and alterations in iron homeostasis have all been shown to confer radioresistance in different tumor contexts. Identifying reliable biomarkers that predict ferroptosis sensitivity will therefore be essential for selecting patients who may benefit from ferroptosis-based therapies.

Second, most available evidence derives from preclinical studies, including cell culture models, xenografts, and experimental RT systems. Although these studies provide valuable mechanistic insights, clinical validation remains limited. Prospective translational research will be required to determine whether ferroptosis-associated molecular signatures correlate with treatment outcomes in patients receiving RT for HNC. Integration of ferroptosis biomarkers into clinical trials may help clarify whether these pathways truly influence patient survival, tumor control, or recurrence risk.

Another important consideration is the potential impact of ferroptosis on normal tissue toxicity. As discussed in the previous section, ferroptosis may contribute not only to tumor cell death but also to radiation-induced injury in normal tissues, particularly in structures such as the salivary glands that are highly sensitive to oxidative damage. Therapeutic strategies designed to induce ferroptosis in tumors must therefore avoid exacerbating ferroptotic injury in surrounding healthy tissues. Achieving this balance will likely require tumor-selective targeting approaches, such as nanoparticle-based drug delivery, metabolic targeting of tumor-specific vulnerabilities, or spatially optimized radiation techniques that minimize exposure of normal structures.

### 7.3. Future Priorities and Translational Directions

Future research directions should focus on several key priorities. First, a deeper understanding of the molecular determinants of ferroptosis competence in HNC is needed, including the roles of lipid metabolism, iron regulation, and redox homeostasis in determining radiation response. Second, the development of clinically applicable ferroptosis-modulating agents will be essential for translating laboratory discoveries into therapeutic interventions. Third, integration of ferroptosis biology with emerging treatment modalities—including immunotherapy, targeted therapy, and particle RT—may provide new opportunities to improve treatment outcomes. Finally, investigation of ferroptosis-based radioprotective strategies may help mitigate treatment-related toxicity and preserve normal tissue function in patients receiving head and neck RT.

Despite growing evidence supporting ferroptosis as a radiosensitization strategy in HNC, several key challenges remain for clinical translation. Future studies should prioritize the use of fractionated radiotherapy models, which more accurately reflect clinical treatment regimens, as most current data are derived from single-dose experimental systems. In parallel, patient-derived organoids and xenograft models will be essential for capturing tumor heterogeneity and validating ferroptosis-targeting strategies in clinically relevant contexts.

Advanced technologies such as spatial transcriptomics and single-cell profiling following RT may further elucidate intratumoral heterogeneity in ferroptosis susceptibility and identify resistant cell populations. The development of non-invasive ferroptosis imaging biomarkers represents another critical need, enabling real-time monitoring of lipid peroxidation and treatment response in vivo. From a therapeutic perspective, rational combination strategies with immunotherapy, particularly immune checkpoint blockade, warrant further investigation given emerging links between ferroptosis and antitumor immunity. Finally, strategies to protect normal tissues, especially salivary glands, from radiation-induced ferroptotic damage will be essential to improve the therapeutic ratio and reduce long-term toxicity.

In summary, ferroptosis has emerged as a critical intersection between oxidative metabolism and RT response in HNC. Experimental evidence indicates that modulation of ferroptosis can influence tumor radiosensitivity, radioresistance, and microenvironmental interactions, highlighting its potential as a therapeutic target. At the same time, the dual role of ferroptosis in tumor control and normal tissue injury underscores the importance of carefully balancing ferroptosis induction with preservation of surrounding healthy tissues. Continued investigation of ferroptosis biology in the context of RT will therefore be essential for translating this promising concept into clinically effective strategies that improve both tumor control and quality of life for patients with HNC.

## Figures and Tables

**Table 1:** Ferroptosis-related mechanisms influencing radiotherapy response in head and neck cancers.

Cancer Type	Molecular Regulator/Target	Main Finding Related to Ferroptosis and Radiotherapy	Reference
HNSCC	Glutamine metabolism/IRF1–TFRC axis	Glutamine blockade enhanced radiotherapy efficacy by inducing immunogenic ferroptosis and increasing intracellular Fe^2+^ levels	[19]
HNSCC	CD47	CD47 inhibition improved macrophage-mediated clearance of ferroptotic tumor cells and enhanced radiotherapy response	[19]
HNSCC	DDR1	DDR1 promoted resistance to carbon ion radiotherapy by suppressing ferroptosis through Akt/mTORC1/SREBP1/SCD1 signaling	[22]
HNSCC	SREBP1–SCD1 lipid metabolism	Lipid desaturation protected tumor cells from ferroptosis and contributed to radiotherapy resistance	[22]
HNSCC	MTDH	MTDH enhanced radiosensitivity by promoting ferroptosis in HNSCC cells	[23]
HNSCC	Ferroptosis-related gene signature	Ferroptosis-associated genes correlated with radiotherapy response and patient prognosis	[20]
HNSCC	MitoTam	MitoTam increased lipid peroxidation and sensitized both radiosensitive and radioresistant HNSCC cells to radiotherapy	[21]
HNSCC	Ferroptosis-related biomarkers	Expression of VDAC1, TFRC, GPX4, SLC7A11, and FTH1 was altered after irradiation, indicating ferroptosis involvement in RT response	[24]
NPC	SLC7A11	Depletion of SLC7A11 increased radiosensitivity and enhanced radiation-induced ferroptosis	[25]
NPC	GSTM3	GSTM3 promoted radiosensitivity through ferroptosis induction via the USP14/FASN/GPX4 axis	[32]
NPC	TXNIP	TXNIP enhanced radiosensitivity by suppressing the xCT–GSH–GPX4 pathway and promoting ferroptosis	[30]
NPC	CD38	CD38 stabilized SLC7A11 and promoted radioresistance by inhibiting ferroptosis	[27]
NPC	METTL3-mediated m6A modification	METTL3 enhanced SLC7A11 expression and inhibited ferroptosis, promoting radiotherapy resistance	[26]
NPC	ACSL4 acetylation	HAT1/HDAC2-mediated acetylation of ACSL4 increased ferroptosis and radiosensitivity	[33]
NPC	circADARB1	Nanocarrier targeting circADARB1 enhanced radiosensitivity through ferroptosis induction	[34]
NPC	SOD2	SOD2 depletion increased lipid peroxidation and enhanced radiosensitivity through ferroptosis	[35]
NPC	Angiotensin II–HIF1α–HILPDA axis	Lipid droplet accumulation suppressed ferroptosis and promoted radioresistance	[28]
NPC	HOTAIRM1/FTO/CD44	lncRNA-mediated signaling reduced ferroptosis and contributed to radioresistance	[29]
NPC	PCK2	Downregulation of PCK2 suppressed ferroptosis and enhanced radioresistance	[31]
NPC	High-dose IR response	Ionizing radiation induced ferroptosis through ROS generation and lipid peroxidation	[36]
NPC	RRFERV lncRNA	Ferroptosis induction sensitized radiation-resistant NPC cells associated with RRFERV expression	[97]
NPC	Stem cell-like traits	Itraconazole triggered ferroptosis and radiotherapy response in NPC spheroid stemness	[98]
NPC	Lipid metabolism regulators	Radiation-induced lipid peroxidation contributed to ferroptosis-mediated tumor cell death	[91]
NPC	Iron metabolism markers	Increased Fe^2+^ and lipid peroxidation markers indicated ferroptosis activation after irradiation	[36]
OSCC	Hyperbaric oxygen	Hyperbaric oxygen enhanced X-ray–induced ferroptosis and radiosensitized OSCC cells	[37]
OSCC	Astaxanthin	Astaxanthin synergized with radiation to promote ferroptosis in oral cancer cells	[38]
OSCC	Cold atmospheric plasma	Plasma irradiation induced oxidative stress and ferroptosis-related death signaling in oral carcinoma	[39]
Thyroid cancer	CHAC1	CHAC1 promoted ferroptosis through glutathione degradation and enhanced radiosensitivity	[40]
ATC	YTHDF2	YTHDF2 stabilized SREBF1 and suppressed ferroptosis, contributing to radiotherapy resistance	[41]

Abbreviations: ACSL4, acyl-CoA synthetase long-chain family member 4; Akt, protein kinase B; ATC, anaplastic thyroid cancer; CHAC1, ChaC glutathione-specific γ-glutamylcyclotransferase 1; circADARB1, circular RNA ADARB1; DDR1, discoidin domain receptor 1; FASN, fatty acid synthase; Fe^2+^, ferrous iron; FTH1, ferritin heavy chain 1; FTO, fat mass and obesity-associated protein; GPX4, glutathione peroxidase 4; GSH, glutathione; GSTM3, glutathione S-transferase mu 3; HAT1, histone acetyltransferase 1; HDAC2, histone deacetylase 2; HIF1α, hypoxia-inducible factor 1 alpha; HILPDA, hypoxia-inducible lipid droplet-associated protein; HNSCC, head and neck squamous cell carcinoma; IR, ionizing radiation; IRF1, interferon regulatory factor 1; lncRNA, long non-coding RNA; m6A, N6-methyladenosine RNA modification; METTL3, methyltransferase-like 3; MTDH, metadherin; mTORC1, mechanistic target of rapamycin complex 1; NPC, nasopharyngeal carcinoma; OSCC, oral squamous cell carcinoma; PCK2, phosphoenolpyruvate carboxykinase 2; ROS, reactive oxygen species; RT, radiotherapy; SCD1, stearoyl-CoA desaturase 1; SLC7A11, solute carrier family 7 member 11; SOD2, superoxide dismutase 2; SREBF1, sterol regulatory element-binding transcription factor 1; SREBP1, sterol regulatory element-binding protein 1; TFRC, transferrin receptor; TXNIP, thioredoxin-interacting protein; USP14, ubiquitin-specific peptidase 14; VDAC1, voltage-dependent anion channel 1; YTHDF2, YTH N6-methyladenosine RNA binding protein 2; xCT, cystine/glutamate antiporter encoded by SLC7A11.

**Table 2:** Comparative ferroptosis-related features across head and neck tumor types.

Tumor Type	Typical RT Sensitivity	Ferroptosis-Related Traits	Key Targets/Pathways
HPV-positive HNSCC	Generally radiosensitive	Lower antioxidant buffering; enhanced immune-associated ferroptosis; increased iron handling	IRF1–TFRC axis, CD47, immune-mediated ferroptosis
HPV-negative HNSCC	Relatively radioresistant	Strong antioxidant defense; lipid remodeling toward MUFA; metabolic adaptation	SLC7A11–GSH–GPX4 axis, SREBP1–SCD1, DDR1, MTDH
Nasopharyngeal carcinoma (NPC)	Highly radiosensitive but prone to acquired resistance	Ferroptosis suppression contributes to radioresistance; strong metabolic and epigenetic regulation	SLC7A11, METTL3, CD38, TXNIP, ACSL4, HIF-1α–HILPDA
Oral squamous cell carcinoma (OSCC)	Intermediate; variable resistance	Intrinsic susceptibility to ferroptosis; oxidative stress–driven lipid peroxidation; immunogenic ferroptosis	GPX4, ROS–lipid peroxidation pathways, ferroptosis inducers
Differentiated thyroid cancer (DTC)	Generally radiosensitive (less RT-dependent clinically)	Ferroptosis linked to glutathione depletion and redox imbalance	CHAC1, GSH metabolism
Anaplastic thyroid cancer (ATC)	Highly radioresistant	Strong lipid metabolic adaptation; ferroptosis suppression via MUFA synthesis	SREBF1–SCD1, YTHDF2-mediated lipid remodeling

## Data Availability

No new data were created or analyzed in this study. Data sharing is not applicable to this article.

## Abbreviations

ACSL4, acyl-CoA synthetase long-chain family member 4; Akt, protein kinase B; ATM, ataxia–telangiectasia-mutated; BH_4_, tetrahydrobiopterin; CAFs, cancer-associated fibroblasts; CoQ_10_, coenzyme Q_10_; DDR1, discoidin domain receptor 1; DHODH, dihydroorotate dehydrogenase; DTC, differentiated thyroid cancer; Fe^2+^, ferrous iron; FSP1, ferroptosis suppressor protein 1; FTH1, ferritin heavy chain 1; FTO, fat mass and obesity-associated protein; GPX4, glutathione peroxidase 4; GSH, glutathione; GSTM3, glutathione S-transferase mu 3; HIF-1α, hypoxia-inducible factor 1 alpha; HNC, head and neck cancer; HNSCC, head and neck squamous cell carcinoma; IR, ionizing radiation; IRF1, interferon regulatory factor 1; LPCAT3, lysophosphatidylcholine acyltransferase 3; m^6^A, *N*^6^-methyladenosine; MDA, malondialdehyde; METTL3, methyltransferase-like 3; mTORC1, mechanistic target of rapamycin complex 1; MUFA, monounsaturated fatty acid; NPC, nasopharyngeal carcinoma; NRF2, nuclear factor erythroid 2-related factor 2; OSCC, oral squamous cell carcinoma; PCK2, phosphoenolpyruvate carboxykinase 2; PUFA, polyunsaturated fatty acid; ROS, reactive oxygen species; RT, radiotherapy; SCD1, stearoyl-CoA desaturase 1; SLC7A11, solute carrier family 7 member 11; SREBP1, sterol regulatory element-binding protein 1; TFRC, transferrin receptor; TXNIP, thioredoxin interacting protein; VDAC1, voltage-dependent anion channel 1; xCT, cystine/glutamate antiporter.
